# Immunotherapy and Microbiota for Targeting of Liver Tumor-Initiating Stem-like Cells

**DOI:** 10.3390/cancers14102381

**Published:** 2022-05-12

**Authors:** Keigo Machida, Stanley M. Tahara

**Affiliations:** Southern California Research Center for ALPD and Cirrhosis, Department of Molecular Microbiology and Immunology, Keck School of Medicine, University of Southern California, 2011 Zonal Ave., 503C-HMR, Los Angeles, CA 90033, USA; stahara@usc.edu

**Keywords:** cancer stem cell (CSC), tumor-initiating stem-like cells (TICs), hepatocellular carcinoma (HCC), immunotherapy

## Abstract

**Simple Summary:**

Hepatocellular carcinoma (HCC) remains one of the more incurable diseases. Thus, finding an HCC treatment is urgent for this unmet medical need. Immunotherapy is a break-through treatment that may help 15–20% of HCC patients. In this review, pharmacological and immune-therapeutical targeting of druggable cancer drivers, immune checkpoints, and long non-coding RNAs for HCC and cholangiocarcinoma are discussed.

**Abstract:**

Cancer contains tumor-initiating stem-like cells (TICs) that are resistant to therapies. Hepatocellular carcinoma (HCC) incidence has increased twice over the past few decades, while the incidence of other cancer types has trended downward globally. Therefore, an understanding of HCC development and therapy resistance mechanisms is needed for this incurable malignancy. This review article describes links between immunotherapies and microbiota in tumor-initiating stem-like cells (TICs), which have stem cell characteristics with self-renewal ability and express pluripotency transcription factors such as NANOG, SOX2, and OCT4. This review discusses (1) how immunotherapies fail and (2) how gut dysbiosis inhibits immunotherapy efficacy. Gut dysbiosis promotes resistance to immunotherapies by breaking gut immune tolerance and activating suppressor immune cells. Unfortunately, this leads to incurable recurrence/metastasis development. Personalized medicine approaches targeting these mechanisms of TIC/metastasis-initiating cells are emerging targets for HCC immunotherapy and microbiota modulation therapy.

## 1. Introduction

Treatment options for HCC are limited. The 3- or 5-year survival rate of HCC is 13–21% and 5%, respectively, without any curative treatment in advanced countries such as the U.S. [1,2,3,4,5]. The incidence rate of extrahepatic metastasis is 13% at 5 years [6,7]. Liver resection is the only viable option for HCC combined with cirrhosis that is the terminal stage of fibrosis, leading to hyperplasia formation [8]. Currently, only 10–23% of HCC patients are candidates for surgery [9,10,11]. Thus, HCV-associated HCC remains an incurable malignancy and an urgent unmet medical need. As 40% of HCCs are derived from TICs, TIC-mediated HCC development is also clinically important.

TICs are resistant to conventional chemotherapy and immunotherapy and persist as recurrent tumors or circulating tumor cells (CTC) [12]. TICs express a core pluripotency-associated transcription factor (TF) network [13,14]. Forty percent of HCCs have clonality and are considered to originate from progenitor/stem cells [15,16,17,18]. TICs express stemness genes that are also expressed in pluripotent stem cells, including CD133 (Prominin in mice), Wnt/β-catenin, Nanog [19], NOTCH, Hedgehog/SMO, BMI, OCT3/4 [20,21,22,23,24,25,26,27,28,29,30,31], CD44 (cell adhesion molecule), and CD34. CD133+/CD49f+ HCC TICs confer resistance to chemotherapy, which hampers efficacy of therapy in HCC [32]. TICs exhibit a loss of this intrinsic asymmetry, leading to subsequent unchecked expansion of the progenitor cell pool [33,34,35,36,37,38]. Cell-fate-determinant molecule NUMB, and p53-MDM2-associated proteins, are targeted by interacting protein TBC1D15 in TICs [39]. These stemness factors are commonly expressed in TICs and pluripotent stem cells. Stemness factors promote therapy resistance and self-renewal ability.

### 1.1. Challenge in Targeting of Actionable Mutations

There are no current targeted therapy options for the most prevalent mutations (most are not “actionable”). HCC has 2.5% of actionable mutations that can be clinically targeted by FDA-approved drugs, while biliary cancer has 45% actionable mutations based on Oncokb.org (Level 3A, Level 3B) and HCC tumor genetics in a TCGA cohort [40]. These indicate many HCC mutations do not have conventional therapeutic targets. Therefore, the role of immunotherapy for the treatment of these diseases is an area of intense investigation [40].

### 1.2. Molecular Tumor Board (MTB) Review and Actionable Mutations in Liver Cancer

The molecular tumor board (MTB) review can guide choices of therapy for actionable mutations, clarify diagnosis, and identify patients who require germline testing. Prospective clinical sequencing of 10,000 patients revealed the mutational landscape of metastatic cancer [41] Clinical actionability of somatic alterations revealed by MSK-IMPACT was the lowest in HCC mutations at 2.5% [41,42,43,44,45], while clinical actionability of somatic alterations revealed by MSK-IMPACT showed that 45% of biliary cancer mutations are clinically actionable. The molecular tumor board (MTB) for intrahepatic cholangiocarcinoma (iCCA) shows clinically targetable mutations [46,47,48]. iCCA is a heterogeneous disease with several identifiable genetic driver mutations (i.e., FGFR2-fusions IDH mutations, etc.) [40]. For CCA, fluorescence in situ hybridization (FISH), DNA/RNA-seq, and immunohistochemistry (IHC) analyses can identify cancer-driver mutations, including IDH1/2, CDK4/67, PRKACA/B, and BRCA1/2. FGFR2 and NYRK fusions, BRAF and IDH1 mutations, and microsatellite instability high (MSI-H)/dMMR (defective mismatch DNA repair) predict responses to targeted/immune therapies [41]. Tumor next-generation sequencing (NGS) should be considered in selected HCC patients with atypical histology/diagnostic features or who may be eligible for clinical trials. HCC classification, cells of origin, genetic and epigenetic abnormalities, molecular alterations, biomarker discovery, and treatments of CCA have been well characterized [49]. The utility of this presumes the detection of actionable targets. Large basket trials showed dramatic responses and long-lasting effects. Therefore, iCCA specimens should be sequenced to identify targetable mutations which have therapeutic implications [40].

## 2. Actionable Targets for Chemotherapies and Immunotherapies

### 2.1. Current Chemotherapy and Immunotherapy Targeting of HCCs 

The frontline drug (Lenvatinib; Table 1) and the second line tyrosine kinase inhibitors (regorafenib, cabozantinib, and ramucirumab; Table 1) improve clinical outcomes (median overall survival remains ~1 year) [41]. Immune checkpoint inhibitors have been widely used for HCC treatment, while combinations of molecularly targeted therapies with immunotherapies are emerging as tools to boost the immune response [40].

### 2.2. Current Immunotherapy for HCC and iCCA 

#### 2.2.1. Anti-PD-1 

Nivolumab (Merck; Table 1) tumor immunotherapy directed at PD-1 was approved by the FDA for advanced HCCs in 2018 [51,68]. Nivolumab showed a manageable safety profile and acceptable tolerability (Table 1; name of trial: CheckMate 459). The objective response rate was 20% (95% CI 15–26) in patients treated with 3 mg/kg Nivolumab and 15% (95% CI; 6–28) in the dose-expansion phase [68]. Pembrolizumab (1 Q3W + BSC 8.3%; Table 1) [62] is currently under Phase III clinical trial (Table 1, Top).

#### 2.2.2. Anti-PD-L1

Anti-PD-L1 [Atezolizumab, Durvalumab, and Ramucirumab (in advanced HCC with AFP > 400 ng/mL); Table 1] are under clinical trials, especially combined with other therapies such as anti-VEGF antibody, including NCT03434379 [50].

#### 2.2.3. Combining VEGF Inhibition and PD-1/PD-L1

Bevacizumab (anti-VEGF) is an anti-angiogenic agent with additional immuno-modulatory effects that decrease the activity of immune-suppressive cells (MDSCs and Tregs) [69,70]. Bevacizumab normalizes tumor vasculature to increase T cell infiltration. Atezolizumab promotes T cell activation by allowing B7.1 co-stimulation. In combination with Atezolizumab (Table 1), Bevacizumab may further reverse VEGF-mediated immunosuppression to promote T cell infiltration into the tumor and enhance Atezolizumab’s efficacy (Table 1). There is a Phase Ib study of Lenvatinib (multi-kinase inhibitor; Table 1) plus pembrolizumab in patients with unresectable HCC [71].

#### 2.2.4. Anti-CTLA-4

Tremelumimab and ipilimumab have been clinically tested [72,73]. The anti-CTLA4 antibody, ipilimumab, increases survival for patients with metastatic cancer, for which conventional therapies have failed. Immunity and immunosuppression regulate anti-tumor immune responses together with the advent of targeted therapies (Table 1). Immunotherapy has been shown to make a durable and long-lasting response in cancer patients [72].

## 3. CCA Treatments under Clinical Investigation

Tumor next-generation sequencing (NGS) should be performed in all advanced Intrahepatic cholangiocarcinoma (iCCA), patients since 45% of driver mutations are actionable in iCCA while only 3.5% of driver mutations are actionable for HCCs from the MSK-IMPACT database [41]. There are several unmet clinical needs for iCCA and HCC targeting, including prolonged durable response, off-label therapeutic risks, and liver dysfunction. Isocitrate dehydrogenase (IDH1) of the Krebs cycle oxidatively decarboxylates isocitrate (ICT) to 2-ketoglutarate (2KG, α-ketoglutarate) to convert NADP^+^ to NADPH and the reverse reaction, i.e., reductive carboxylation of 2KG to ICT that oxidizes NADPH to NADP^+^ [40,41]. Gain of function mutations make neomorphic IDH1, and two alleles produce oncometabolite 2-hydroxyglutamate (2-HG) (Table 2). Ivosidenib and Larotrectinib target the IDH1 mutant CCA [40,41].

Pemigatinib targets FGFR fusions and works for CCA (Table 2). Fisogatinib targets FGF19+ cancers (Table 2). Larotrectinib had marked and durable antitumor activity in patients with TRK fusion (+) cancer, regardless of the age of the patient (both adults and children) or of the tumor type (ClinicalTrials.gov numbers, NCT02122913, NCT02637687, and NCT02576431) [74].

### 3.1. Bacterial Species That Are Associated with Responsiveness to Immune Checkpoint Inhibitors

**Figure 1:**
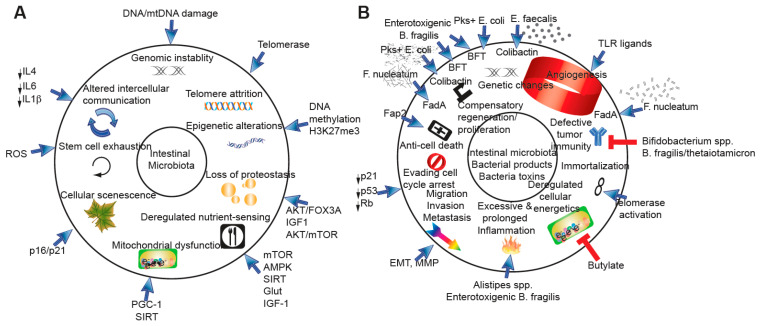
Intestinal microbiota and bacterial products. (**A**) Nine hallmarks of intestinal microbiota effects on host. (**B**) Summary of intestinal microbiota bacterial products, including bacterial toxins and their effects on the host.

Several bacterial species are associated with improved responses against immune checkpoint inhibitors. The modulation of gut microbiota improves the efficacy of PD-L1 blockage therapy [78]. Bacterial species associated with positive responses to PD-1 and PD-L1 blockade therapy are summarized in Figure 1. Thus, the microbial community is effective as a co-therapy. Notably, fecal transplants have been shown to inhibit tumor growth (Figure 1).

### 3.2. PD-1 Antibody (Nivolumab)

**Figure 2:**
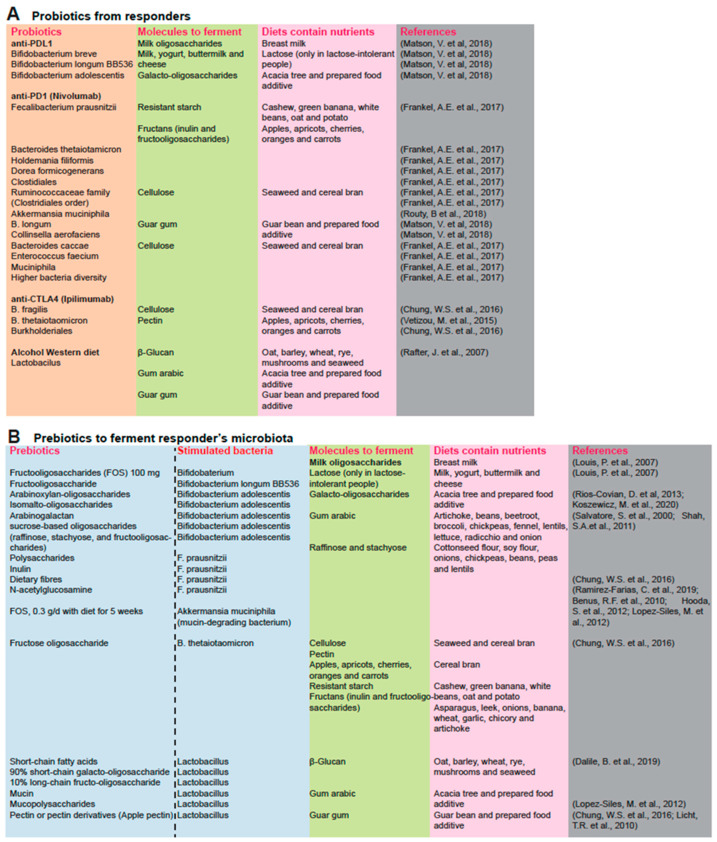
Probiotics and prebiotics fermented by beneficial bacteria for immune checkpoint inhibitors. (**A**) Bacterial species that are positively associated with PD-1 and PD-L1 blockade therapy are summarized. Responders for immunotherapy have specific gut microbiota. Bacteria species are summarized in a table from the literature [20,79,80,81,82,83,84,85,86,87,88,89,90,91,92,93,94,95,96,97,98,99,100,101,102,103,104,105]. (**B**) Bacterial species and prebiotics that are positively associated with PD-1 and PD-L1 blockade therapy are summarized.

Probiotics exert an adjuvant effect to enhance the anti-PD-1 response (Figure 2A), including: *Fecalibacterium prausnitzii*, *Bacteroides thetaiotamicron*, *Holdemania filiformis*, *Dorea formicogenerans*, *Clostridiales* (indigenous Clostridia induce Treg accumulation, presumably by cooperating with DC in the colon [79]), *B. longum*, *Collinsella aerofaciens*, *Enterococcus faecium*, *Ruminococcaceae*, *Muciniphila*, *Faecalibacterium*, *Enterococcus hirae*, and *Bifidobacterium*. In addition, *Akkermansia muciniphila* was enriched in patients who responded to anti-PD-1 therapy [80]. This suggests that *A. muciniphila* may enhance patient response to PD-1 blockade therapy [80]. Cancer patients who responded to immune checkpoint inhibitors that were enriched with *Bacteroides caccae* detected by the metagenomic shotgun sequencing method [81], which are antibody dependent. Patients who responded to anti-PD-1 therapy exhibited a higher bacterial diversity. Patient microbiota who responded to nivolumab (PD-1 antibody) had increased gut bacterial species, including: *Fecalibacterium prausnitzii*, *Bacteroides thetaiotamicron*, and *Holdemania filiformis*. On the other hand, patient gut microbiota who responded to pembrolizumab (another PD-1 antibody) had an abundance of bacteria from the *Ruminococcaceae* family and *Dorea formicogenerans* when compared to patients who did not respond to this therapy (Figure 2A). Germ-free mice transplanted with fecal samples from patients responding to anti-PD-1 and anti-PD-L1 therapy showed a reduction in tumor growth and improved responses to anti-PD-1 and anti-PD-L1 therapy. These mice also showed a higher density of CD8+ T cells [82]. *B. longum*, *Collinsella aerofaciens*, and *Enterococcus faecium* were observed to be more abundant in the anti-PD-1 immunotherapy responders, supporting the anti-tumor effects of *Bifidobacterium* species [83]. *Bifidobacteriaceae* and *Erysipelotrichaceae* are increased in PD-1 therapy responder patients (Figure 2A). Thus, the gut microbiome influences the efficacy of PD-1-based immunotherapy against epithelial tumors with DNA repair proficiency [80]. Taken together, studies show less overlap between bacterial species that are enriched in responder patients compared to non-responders. Further studies are warranted to study this question.

### 3.3. Anti-PDL1 Antibody

Bifidobacterium species enhance protective immunity against tumors, including *Bifidobacterium breve* [83], *Bifidobacterium longum*, and *Bifidobacterium adolescentis* [83] (Figure 2).

### 3.4. Anti-CTLA4 Antibody

Dietary fibers such as inulin stimulate the human-specific species (e.g., *F. prausnitzii* population; Figure 2B) of gut microbiota [84]. The *Bacteroides* species influence the antitumor effects of the CTLA-4 blockade. For example, the T cell responses specific for *B. thetaiotaomicron* or *B. fragilis* were associated with the improved efficacy of CTLA-4 blockade in both human patients and mice [84]. CTLA blockade treatments were ineffective in antibiotic-treated or germ-free mice [85]. A CTLA4 response could be achieved by gavage with *B. fragilis*, by inoculation with *B. fragilis* polysaccharides, or by adoptive transfer of *B. fragilis*-specific T cells in order to overcome any non-responsive issues associated with CTLA4 blockade [85]. The outgrowth of *B. fragilis* following fecal microbial transplantation from patients to mice confirmed the observed response to CTLA-4 blockade therapy in melanoma patients [85]. *Bacteroidales* spp. modulate the immune reactions in response to CTLA-4 blockade (Figure 2). Fecal microbial transplantation from humans of other *Bacteroidales* to mice restored a therapeutic anti-cancer response to anti-CTLA-4 as shown by *Bacteroides fragilis* [Bf], *Burkholderia cepacia*, and *B. thetaiotaomicron*. These species improved regulatory T (Treg) cell differentiation mediated by PSA-activated DC [85].

Bacteroides spp. (for example, *Bacteroides fragilis* [Bf]) promote responses to CTLA-4 blockade immunotherapy [85,106]. Fecal microbial transplantation (FMT) enhanced antibodies against CTLA-4 and favored the outgrowth of *B. fragilis* with anticancer properties in cancer patients (Figure 2). *Bacteroidales* stimulate the immune responses of the CTLA-4 blockade [85].

### 3.5. CpG-Oligodeoxynucleotides (ODNs) Activate DCs to Clear Tumors

Unmethylated CpG motifs stimulate the immune system through pathogen-associated molecular patterns (PAMPs) [86] consistent with their abundance in microbial genomes but their rarity in vertebrate genomes [87]. Therefore, *Alistipes* and *Ruminococcus* assist CpG-ODNs to activate DCs in order to clear tumors, since the pattern recognition receptor (PRR) Toll-Like Receptor 9 (TLR9) recognizes the CpG PAMP [87] and is expressed in B cells and plasmacytoid dendritic cells (pDCs) in humans and other primates [87].

## 4. Potential Therapeutic Strategies by Fecal Microbiota Transplantation, Probiotics, and Prebiotics

The bacteria of the bacteriome (10^13^–10^14^), the fungal mycobiome (10^12^–10^13^), helminths (multicellular eukaryotes: 0–10^4^), and members of the virome (10^14^–10^15^) [88] together colonize humans and other animals to form the gut microbiota. These share the same host niches and must compete, antagonize, synergize, or interact among themselves and their host [88]. Examples of such interactions include: (i) beneficial bacteria acting as drugs (dysregulated microbiota can be restored by FMT); (ii) bacteriome alterations by phages can restore the healthy gut microbiota and antibiotic and/or antifungal treatment may restore healthy gut microbiota; and (iii) drugs produced by bacteria gut barrier stabilization changes can occur by microbial bile acid metabolizing enzymes that change primary bile acids to secondary bile acids from FXR to FGF10. Therefore, FGF10 analogues or FXR agonists can be drug targets for gut barrier stabilization changes to the microbial bile acid metabolizing enzymes [89,90].

### 4.1. Nutritional Interventions and Diet Therapy

Fecal enzyme-linked immunosorbent assay shows that α-1-antitrypsin levels can distinguish cholangiocarcinoma patients from normal individuals. Therefore, α-1-antitrypsin level is a potential marker for early diagnosis of cholangiocarcinoma [91].

#### Therapeutic Effects of Mediterranean Diet on NAFLD/NASH

The Mediterranean diet contains beneficial nutrients which may counter nonalcoholic fatty liver disease (NAFLD)/nonalcoholic steatohepatitis (NASH) and can contribute to longevity by inclusion of ω-3-fatty acids, polyunsaturated fatty acids antioxidants, and choline [91]. Individuals that live longer are accustomed to eating foods with particular beneficial molecules, including soybeans (flavones), seaweed (minerals), seafood (fish oils: Docosahexaenoic acid (DHA), ω-3 fatty acids (eicosapentaenoic acid (EPA), butylated hydroxyanisole (BHA)), and green tea without sugar (polyphenols, and catechin epigallocatechin-3-gallate: EGCG) [91].

### 4.2. Specific Nutrients of Diets Stimulate Specific Bacteria

Nutritional interventions (dietary fibers) with prebiotic inulin intake stimulates *Faecalibacterium prausnitzii* of the human gut microbiota [84,92]. The latter consumes polysaccharides of the gut lumen (such as arabinogalactan, xylan, and soluble starch; Figure 2B) [7], cellulose, and laminarins from seaweeds and can grow on apple pectin and pectin derivatives [20,93].

Various prebiotic treatments (pectin or pectin derivatives and *N*-acetylglucosamine) stimulate the beneficial gut bacteria *F. prausnitzii* in healthy human volunteers [92,94,95]. Apple pectin feeding promotes *Firmicutes* growth in rats [96], since pectin or pectin derivatives stimulate *F. prausnitzii* growth [84] which can compete with other bacteria for pectin utilization [97]. Accordingly, *F. prausnitzii* encodes pectinolytic enzymes [107]. *F. prausnitzii* strains can utilize the glycoprotein *N*-acetylglucosamine [97] found in gut mucosa [98]. Treatment with *N*-acetylglucosamine heals inflamed and damaged soft tissues of the gut [98], and restores gut function to improve Crohn’s disease (CD). Mucin stimulates growth of the beneficial bacteria *F. prausnitzii* [99], since *F. prausnitzii* isolates cannot utilize mucin or mucopolysaccharides [97]. *F. prausnitzii* switches between substrates derived from the diet or the host and benefits from mucin metabolism (Figure 2). Therefore, diet-derived nutrients can facilitate gut damage repair via beneficial bacterial growth, such as by *F. prausnitzii* (Figure 2B).

### 4.3. Treatment of Probiotics, Prebiotics, and Synbiotics Promote the Beneficial Bacteria Growth

Probiotics and prebiotics synergistically combine to heal the leaky gut and promote better immune responses. For example, combination of prebiotic inulin and probiotics *Bifidobacterium lactis* Bb12 and *Lactobacillus rhamnosus* GG decreased the growth of *Clostridium perfringens*, but increased *Bifidobacterium* and *Lactobacillus* as detected in fecal microbiota (Figure 2B). Apparently, this improved epithelial barrier function in patients diagnosed with colonic polyps, decreasing growth of the latter.

Dietary synbiotics reduce cancer risk factors in polypectomized and colon cancer patients [100]. This was shown by treatment with combinations of probiotics and prebiotics (called synbiotics) including the ingredients: group S (*Lactobacillus acidophilus* 10, 1 × 10^9^ CFU, *Lactobacillus rhamnosus HS 111*, 1 × 10^9^ CFU, *Lactobacillus casei* 10, 1 × 10^9^ CFU, *Bifidobacterium bifidum*, 1 × 10^9^ CFU, and fructo-oligosaccharides (FOS) 100 mg) compared to placebo–control group C (Figure 2B). Treatments were given twice daily, for a total of 14 days and found to be beneficial [100]. Furthermore, *Lactobacillus acidophilus* NCFM facilitates mouse myeloid dendritic cells (DCs) to express antiviral genes, such as myxovirus resistance 1, IFN-β, and IFN stimulated via the TLR2 pathway [100].

### 4.4. Antibiotic Treatment with Fecal Microbiota Transplantation (FMT) Ameliorates Alcoholic Liver Disease (ALD)

#### 4.4.1. Alcoholic Hepatitis Patients Have Dysbiotic Gut Microflora with Marked Loss of Butyrate Producers

Targeting IL-17 signaling is a novel therapeutic strategy for ALD. Alcoholic hepatitis (AH) patients have dysbiotic gut microbiota with the marked loss of butyrate producers, but with the increased serum and hepatic IL17 [11]. IL-17 signaling regulates the liver–brain axis and intestinal permeability in ALD [102]. ALD mouse models resulting from ethanol feeding do not show increased IL17A nor T cell inflammatory responses [11], and therefore do not fully replicate human severe alcohol-associated liver diseases (Figure 2). Alcohol-induced dysbiotic gut microbiota and/or their products drive T-cell-specific IL17 responses that are pathogenic in human AH [11].

Microbial involvement in AH could be demonstrated by bi-weekly human AH-FMT to C57Bl/6 mice, which led to loss of butyrate-producing bacterial families with decreased butyrate levels in cecal samples [11]. AH-FMT increases hepatic and plasma IL17A levels regardless of alcohol exposures. The Th17 and γδT cells are increased by AH-FMT. IL17A + CD4 cells also increase after AH-FMT with increases in hepatic inflammatory cytokines, chemokines, hepatic steatosis, and injury [11].

The AH-FMT model recapitulates hepatic pro-inflammatory T cell responses observed in AH patients [11], including increased hepatic Th17, γδT cells, increased hepatic and plasma IL17A levels, but decreased hepatic Tregs [11].

Therapeutic IL-17 targeting showed improvements in three different ALD mouse models: (1) an intragastric ethanol feeding model that recapitulated alcoholic steatohepatitis and fibrosis; (2) a chemical carcinogen diethylnitrosamine (DEN) + alcohol model that mimicked liver cancer associated with alcohol misuse; and (3) a chronic feeding with weekly binge drinking model that mimicked alcoholic hepatitis and steatohepatitis (Figure 2). It is the dysbiotic gut microbiome that causes pathology of alcohol-induced hepatitis [11]. However, most effects did not require ethanol feeding [11]. Fifteen-day AH-FMT treatment resulted in expression of the defensin-resistant multiple peptide resistance factor (*MprF*) protein which counteracts the effects of defensins expressed in the gut (Figure 2). MprF consists of separable domains for lipid lysinylation and antimicrobial peptide repulsion [101]. Anti-cytokine therapy is used for rheumatoid arthritis and dysbiosis associated with Crohn’s disease. It is also used in alcoholic liver disease, with inhibition of the deleterious effects of these potential cytokine storms.

#### 4.4.2. NASH and Cancer Patients

Patients post-fecal microbiota transplant (FMT) had lower abundance of vancomycin (VanH), β-lactamase (ACT), and the rifamycin antibiotic-resistance gene (ARG); this was associated with cognitive improvement [24]. Pre-FMT antibiotics for these patients included metronidazole 400 mg (TDS), ciprofloxacin 500 mg orally (BD), and amoxicillin 500 mg orally (TDS). All antibiotic treatments were discontinued 12 h before FMT. In the antibiotics + enema trial for post-antibiotics at day 7 vs. baseline, vancomycin and β-lactamase ARGs were elevated and decreased at day 15 [24]. Between standard-of-care (SOC) and FMT, after seven days lower levels of ARG (cfxA β-lactamase, VanW, and VanX) was observed, since ciprofloxacin (cfxA) targets a class A β-lactamase found in *Bacteroides vulgatus* [24]. These ARGs are markers for changes in the bacteriome. ARG abundance is largely reduced after FMT in decompensated cirrhosis [24].

#### 4.4.3. Participants in the Standard-of-Care (SOC) Group Did Not Receive Pre-Therapy Antibiotic

Fecal microbiota transplant from a rational stool donor improved hepatic encephalopathy in a randomized clinical trial [25]. Post-antibiotics, beneficial taxa, and microbial diversity reduction was observed with proteobacteria expansion [25]. However, normal FMT increased diversity and beneficial taxa [25]. The standard-of-care (SOC) microbiota and model for end-stage liver disease (MELD) score [108] remained similar throughout. Thus, FMT from a selected rational donor reduced hospitalizations and improved cognition and dysbiosis in subjects with cirrhosis with recurrent hepatic encephalopathy (HE) [25].

### 4.5. Endogenous Retrovirus Activation Turns on IFN Signaling Pathways to Activate Immunotherapy-Mediated CTL

Epigenetic regulators turn on endogenous retroviruses that activate cGAS-cGAMP-STING-mediated cytosolic DNA sensing and IFN signaling [26]. To activate immune checkpoint inhibitor mediated CTLs, endogenous retrovirus-mediated lncRNA activates RNA sensor RIG-I pathways to induce type I IFN signaling pathways [27]. Highly conserved endogenous retroviral elements (ERVs)-lncRNA are activated in numerous cancers [27]. Tumors with constitutive activation of endogenous retrovirus become resistant to chemotherapy and immunotherapies. Moderate to high levels of endogenous retroviral-associated adenocarcinoma RNA, or ‘EVADR’, were detected in 25 to 53% of colon, rectal, lung, pancreatic, and stomach adenocarcinomas. EVADR expression correlates with decreased patient survival [27]. Therefore, inhibiting an endogenous retrovirus may promote the efficacy of immune checkpoint inhibitors [27].

### 4.6. Metabolites from Gut Microbiota Produce Bile

Metabolites from gut microbiota pass through the gut epithelial layer and reach the liver through the portal vein and affect bile production [89,90]. Alcohol increases bile acids but reduces short-chain fatty acids in the gut [89]. Heavy alcohol intake and/or excessive intake of fat and/or fructose induces inflammasome-mediated dysbiosis to promote ALD and NAFLD in Western countries [28,29]. Microbes and their metabolic products promote liver disease. Identification of microbial biomarkers of HCCs and treatment to manipulate the gut microbiota are an emerging field. Analysis of the intestinal microbiome of HCC patients will allow selection of specific microbiota-based probiotics or FMT therapies.

Apical sodium-dependent bile acid transporter (ASBT, known as ileal bile acid transporter (IBAT) and SLC10A2) inhibition for 16 weeks improved multiple features of NASH in a high unsaturated fat diet (HFD) mouse model [30]. Inhibition of ileal bile acid uptake protects against NAFLD in high fat diet fed mice [30]. ASBT inhibition restored glucose tolerance and reduced hepatic triglyceride and total cholesterol concentrations, which improved NAFLD activity scores in HFD-fed mice. [30] Interruption of the enterohepatic bile acid (BA) circulation further protects against NAFLD [30]. Blocking ASBT function with a luminally restricted inhibitor also improves NAFLD [30].

### 4.7. Mechanism of Fecal Microbiota Transplantation through Immune Systems

Transplantation of fecal microbiota from alcoholic hepatitis patients induces hepatic recruitment of IL-17-producing inflammatory T cells promoting inflammation and injury [11]. Alcoholic hepatitis patients have unique characteristics of dysbiotic gut microbiota, including (i) loss of biodiversity, (ii) loss of total microbial numbers, (iii) loss of specific microbial population of metabolites, and (iv) relative enrichment of other bacterial exotoxin A (ETA: ToxA)/increased metabolites [11].

## 5. Bacterial Metabolites

### 5.1. Nutrients Maintain Gut Integrity

Conversion of starches to short-chain fatty acid (SCFA) maintains gut integrity [31]. Dietary-derived substrates (e.g., apple pectin, seaweed cellulose and laminarins, oat β-glucan) ferment to maintain beneficial bacteria. Short-chain fatty acids mediate an interplay between diet, gut microbiota, maintenance of gut integrity [20], and host energy metabolism [36].

### 5.2. The Role of Short-Chain Fatty Acids in Microbiota–Gut–Brain Communication

Short-chain fatty acids (SCFAs), the main metabolites produced by bacterial fermentation of dietary fiber in the gastrointestinal tract, are speculated to have a key role in microbiota–gut–brain crosstalk. Short-chain fatty acids (SCFAs) can mediate microbiota–gut–brain axis crosstalk [20] through interaction with G-protein-coupled receptors or histone deacetylases and regulation of direct humoral effects, which are indirect hormonal and immune pathways [37,38]. Dietary intervention regulates cognition and emotion through the gut–brain axis via SCFAs [37,38]. SCFAs should be quantified in the systemic circulation in dietary intervention studies, in which the effects on psychological functioning and psychopathology are an outcome of interest [37,38].

## 6. Gut-Microbiota-Mediated Immune Regulatory Mechanisms by Immunotherapy

The microbiome is a therapeutic target for numerous cardiometabolic disorders by drugging the microbiome [43]. Bacteria from specific microbes are associated with diagnosis of colorectal cancer. Some intestinal microbiota promote colorectal carcinogenesis. Clinicians should evaluate patients with bacteremia from specific bacteria for cancer lesions in the colorectum.

### 6.1. Bacterial Enzyme Inhibitors Can Be Used for Treatment

Gut microbial produced metabolites can be recognized by host pathogen recognition sensors to promote HCC progression. Metabolism of dietary components by the gut microbiota produces short-chain fatty acids, including other metabolites. When combined with microorganism fragments, these can stimulate the meta-organismal endocrine axis to promote HCC onset and growth. For example, trimethylamine (TMA) produced in the gut promotes ALD [44]. Thus, pharmacological interventions at the level of the gut microbial endocrine organ should reduce HCC risk.

Targeting of the gut microbiota has great potential as a therapeutic modality for many diseases. However, relatively little is known regarding the contribution of commensal bacteria to normal host physiological functions [45]. For example, it was reported that 11 bacterial strains in feces obtained from normal human donors induce CD8 T cells to produce IFN-γ in the intestine in the absence of a generalized inflammation response dependent on CD103+ DC and MHC class Ia [45]. These 11 strains also improved the efficacy of immune checkpoint inhibitors and aided host suppression against *Listeria monocytogenes* infection [45]. Thus, these 11 identified strains, which represent low-abundance components of the human microbiome, are potential biotherapeutics [45].

### 6.2. TLR2 Signaling in DCs Promotes Treg Differentiation to Attenuate the Inflammation

**Figure 3:**
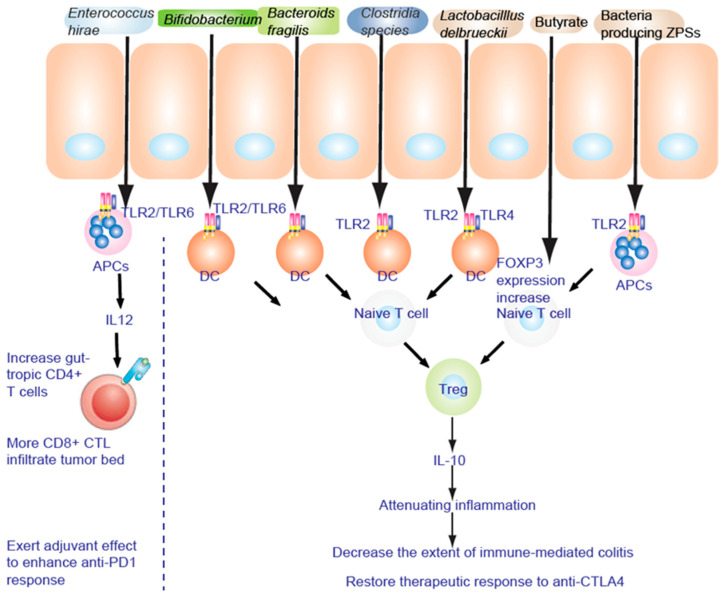
TLR2 is necessary to alleviate the inflammatory response. Fructo-oligosaccharide and inulin are considered as prebiotics, affecting IECs to be hyporesponsive to activation of NF-κB and MAPK induced by pathogens. NF-κB and MAPK reduce the inflammatory response to lipopolysaccharide (LPS).

TLR2 senses components from bacteria, mycoplasma, fungi, and viruses [47] to activate NF-κB to promote a Th17 cell response to enhance the inflammation response and anti-inflammation responses [48,109]. *Lactobacillus acidophilus* stimulates the TLR2 pathway of murine myeloid dendritic cells (mDC) to induce interferon-β (IFN-β), while IL-10 secretion in plasmacytoid DC (pDC) is TLR9 dependent (Figure 3). *Bifidobacterium infantis 35624* stimulates the TLR2/TLR6 pathway to increase IL-10 secretion from human DCs. Polysaccharide A of Gram negative bacilli can activate TLR2 and promote the secretion of anti-inflammatory cytokine IL-10 [110]. These diverse immune responses depend on the appropriate co-receptor and microenvironment [48].

### 6.3. Regulatory T Cells

Treg cells secrete the anti-inflammatory cytokine IL-10 to attenuate inflammation. IL-6, IL-21, and IL-2 dynamically regulate the balance between Th17 and Treg cell differentiation [111,112]. Intestinal bacteria act to stimulate and shape the T cell subsets. Short-chain fatty acid primed and induced Th17 cells undergo differentiation locally in the lamina propria. In addition, segmented filamentous bacteria antigen (SFB) adhesion to enterocytes stimulates serum amyloid A and ROS to induce Th17 cells [113]. MHCII-dependent antigen presentation of SFB occurs on DC [114] (Figure 3). Commensal bacteria (such as the *Lachnospiraceae* family, A4 bacteria) induce transforming growth factor β (TGF-β) production to inhibit Th2 cell development [115]. Clostridia colonization effect on T cell differentiation induces Treg cell expansion to suppress inflammation in mice [79,116]. In germ-free (GF) mice, colonization of gut bacteria and LPS-rich sterile diet induced T and B cell proliferation and differentiation in Peyer’s patches (PP) and mesenteric lymph nodes (MLN), especially by CD4+ Foxp3 + T cells in MLN [117]. Polysaccharides do affect T cell differentiation. To reinforce its intestinal colonization, polysaccharide A (PSA) from *Bacteroides fragilis* promotes Treg cell secretion and suppresses Th17 activity [118]. The growth of bacteria encoding zwitterionic capsular polysaccharides (ZPS), as shown by genomic screen, results in stimulation of T cell differentiation of Treg cells and IL-10 production mediated by antigen presenting cells (APC) [119].

Zwitterionic polysaccharides bind the TLR2 complex on CD11b^+^ DC to mobilize lamina propria CD11b^+^ DC. This in turn stimulates Treg differentiation to promote anergy against immunity induced by CTLA-4 blockade [120] via interleukin-12 (IL-12)-dependent cognate TH1 immune responses against Bf capsular polysaccharides (Figure 3). CTLA4-mediated TH1 immune response is blocked by Treg to protect against experimental abscess formation [120] independent of TLR2/TLR4-mediated innate signaling [121,122]. A clustering of genus composition of stools [123,124] distinguished three clusters with *Alloprevotella or Prevotella* driving cluster A and distinct *Bacteroides* spp. driving clusters B and C. During anti-CTLA4 (ipilimumab) therapy, the proportions of MM patients falling into cluster C increased at the expense of those belonging to cluster B through the colonization of the immunogenic bacteria *Bf* and *Bt* [120,121,122,125,126,127].

### 6.4. Commensal Bacteria-Derived Products Stimulate DCs and Regulate Tregs

High-alcohol-producing *Klebsiella pneumoniae* causes fatty liver disease [128]. Intestinal microbiota in human stool contributes to susceptibility to ALD shown by the use of ALD-FMT in germ-free mice [129,130]. To edit gut microbiota, four distinct bacteriophages (podophages of the virulent *Picovirinae* group) were isolated from sewage water. Feeding of four podophages of the virulent *Picovirinae* group lyse the cytolytic *E. faecalis* strain [131]. Gavage of bacteriophages that target cytolytic *E. faecalis* attenuates alcoholic liver disease that promotes *E. faecalis* expansion (2700-fold increase) by reducing steatosis, inflammation, and liver injury of mice chronically fed ethanol [132]. Therefore, the gut microbiome is a therapeutic target in the pathogenesis (pro-inflammatory response) and treatment of chronic liver disease [133], since it is altered in liver cirrhosis [134]. Overgrowth by Clostridiales, Streptococcus, Lactobacillus, Bacteroides, and Enterobacteriaceae genera promotes gut injury and liver disease. In liver cirrhosis, Bacteroides increase while Firmicutes decrease. Rifaximin inhibits oral-originating species and selectively decontaminates the gut. Further environmental factors mediating microbiota changes can promote excessive inflammatory signaling.

### 6.5. A Live Microbiome Co-Culture in a Gut-on-a-Chip Microfluidic Device

A live microbiome was co-cultured with micro-engineered human intestinal villi in a gut-on-a-chip microfluidic device [135]. The intestine–liver axis on-chip reveals the intestinal protective role on hepatic damage (Figure 3) by emulating ethanol first-pass metabolism [136,137,138,139]. Those who live longer customarily consume the following foods, including pasta (barley: fibers), soybean (flavone), seaweed (mineral), seafood (fish oil: DHA, BHA), and green tea (polyphenols, catechin epigallocatechin-3-gallate: EGCG). Prebiotics are nondigestible dietary supplements, including mucin or long-chain carbohydrates, which promote proliferation of beneficial commensal bacteria and improve the ecological balance of the gut. The effects of prebiotics can be tested in this system (Figure 3).

Synbiotic treatment normalizes gut microbiota and concomitantly reduces toxic gut microbiota to repair leaky guts [105]. These bacteria digest prebiotics to produce short-chain fatty acids which inhibit intestinal pathogen growth, provide enterocyte nutrition (butyrate), and promote mineral absorption. Bifidobacterium growth is enhanced with a prebiotic-containing formula (90% short-chain galacto-oligosaccharide, 10% long-chain fructo-oligosaccharide), fructo-oligosaccharides [103], and inulin [104] (Figure 2).

Patients who responded to nivolumab (PD-1 antibody) were enriched with *Bacteroides caccae* [81] and *Fecalibacterium prausnitzii*, *Bacteroides thetaiotamicron*, and *Holdemania filiformis*, whereas patients who responded to pembrolizumab (another PD-1 antibody) showed that their gut microbiota was enriched with *Dorea formicogenerans*. This treatment increased bacterial diversity and abundance of bacteria from *Akkermansia muciniphila* [80], *Bifidobacterium* spp. (*B. longum*, *Collinsella aerofaciens*) [83], *Enterococcus faecium*, and the *Ruminococcaceae* family, induces Treg accumulation by cooperating with DC in the colon (Figure 3).

### 6.6. TLR2 Is Necessary to Alleviate the Inflammatory Response

TLR2 senses components from bacteria, mycoplasma, fungi, and viruses [47]. TLR2 signaling induces both pro- and anti-inflammation responses. *Bifidobacterium infantis 35624* treatment increases IL-10 secretion through the TLR2/TLR6 pathway in human myeloid dendritic cell (mDC) and monocyte-derived DC (MDDC), while IL-10 secretion in plasmacytoid DC (pDC) is TLR9 dependent. Tregs secrete the anti-inflammatory cytokine IL-10 to attenuate inflammation (Figure 3). Therefore, feeding of FMD + synbiotics preconditions gut microbiota and repairs the leaky gut to improve immunotherapy and chemotherapy (Figure 3).

### 6.7. Metabolism and Local Effects of SCFAs

Fermentation of dietary fiber in the colon generates short-chain (ranging from one to six carbon atoms) saturated fatty acids (SCFAs) [140]. Production of SCFA is dependent on dietary fiber and can result in gut production of approximately 500–600 mmol of SCFAs per day [141]. Acetate (C2) is the most abundant SCFA in the human body, followed by propionate (C3) and butyrate (C4) (in a molar ratio of 60:20:20, dependent on microbiota composition) as the most abundant anions in the colon [142,143]. Bowel movements transfer gut contents from the terminal ileum to the proximal colon where SCFAs can reduce the pH. Lesser amounts of other SCFAs, including caproate, formate, and valerate, are also produced [142]. Monocarboxylate transporters (MCTs) allow SCFA absorption by colonocytes in an H^+^-dependent, electroneutral manner, whereas the electrogenic, sodium-dependent monocarboxylate transporter 1 (SMCT1; known as SLC5A8) transports the SCFA anion [144].

Because of microbiota changes or intestinal microbiota transplantation in liver diseases and cirrhosis, use of pharmacotherapeutics must be cognizant of these issues when considering treatment options [145]. Transfer of intestinal microbiota from lean donors increases insulin sensitivity in individuals with metabolic syndrome [146]. Allogenic fecal microbiota transplantation in patients with NAFLD improves abnormal small intestinal permeability, as shown in a randomized control trial [147]. Alkaline phosphatase can be used as a surrogate marker for liver–gut changes. *C. difficile* (+) cirrhosis is a deleterious combination with greater mortality via brain dysfunction due to SCFA downregulation. GF mice have altered microbial infection inflammatory markers (IL1β, MCP1, and IBA). Post-FMT GF mice recipients show improved neuro-inflammation [148]. Use of capsular fecal transplantation improves microbial function and supports better clinical outcomes in cirrhosis [149]. Oral capsule FMT (containing *Ruminococcaceae*) is currently under investigational new drug application (IND) guidance [150]. A randomized clinical trial of fecal microbiota transplant for alcohol use disorder is ongoing.

### 6.8. Exercise or Phage Therapy Retards Liver Diseases

Exercise reduces the incidence and progression of hepatocellular carcinoma in mouse models [151]. Personalized medicine approaches will stratify the HCC patient population into distinct subpopulations that may be responsive to HCC-type specific treatments [151]. As presented in this review, there are several avenues of liver morbidities leading to HCC. For example, the microbiota is targeted for cytolysin + alcoholic hepatitis patients. Future investigations will support a better understanding of antibiotic therapies for enteric pathogens, long-term effects of phage-based treatments, and precisely editing bacteria genomes by phage therapies (single phage or phage cocktail). These are all emerging areas of investigation, and these options reflect the original intent to reverse the triggering events leading to HCC.

### 6.9. Caveats for Fecal Microbiota Transplantation

FMT with multi-drug-resistant organisms (MDRO) can cause problems in donor recipient patients. Avoidance of *C. difficile* is important since it is responsible for the chronic liver diseases cirrhosis and alcoholic hepatitis. FMT trials for chronic liver diseases are currently in progress.

## 7. Concluding Remarks

Future investigative projects need to address specific treatments and FMT short-term changes in patients with HCC. Another consideration is whether allogeneic or autologous FMT should be employed, especially since current FDA-approved immunotherapies, such as anti-PD-1 or anti-CTLA4, have limited efficacy only for a small fraction of HCC patients (10–25% range undergoing monotherapy). The remaining HCC patients do not respond to this monotherapy, thus other immune mechanisms may be needed to allow synergism with tumor-killing cells, such as antigen-presenting cells, including dendritic cells and B cells. Inclusion of immune checkpoint inhibitors with combination therapy may break immune tolerance and improve the therapeutic efficacy of this approach.

## Figures and Tables

**Table 1:** First line therapy (FDA-approved and Phase III investigations in advanced HCC).

Therapy	Target	OR (HR)	Note	Clinical Phases [40] *	Reference and ClinicalTrials.gov
**Atezolizumab +** **Bevasizumab**	PD-L1, VEGF	0.58 (HR 0.42–0.79)	OS > 17 months, normalizes tumor vasculature to starve tumors	Approved, III	NCT03434379 [50]
**Nivolumab**	PD-1	0.85 (0.72–1.02)	Well-tolerated in advanced HCC	Approved, III (sorafenib)	NCT02576509 [51]
**Lenvatinib**	FGFR1-4, VEGFR1-3, RET, c-KIT and PDGFRα	0.85 (0.72–1.02)	Safety and tolerability profiles	Approved, III non-inferiority trial	NCT01761266 [52]
**Erlotinib + Sorafenib**	EGFR + VEGFR1-3, RET, KIT PDGFRa/b	0.92 (0.78–1.11)	Adding erlotinib to sorafenib did not improve survival	III	NCT0901901 [53]
**Linifanib**	FGFR	1.05 (0.90–1.22)	TTP and ORR favored linifanib	III	[54]
**Brivanib**	VEGFR	1.07 (0.94–1.23)	OS, TTP, ORR, DCR, and mRECIST	III	NCT00858871 [55]
**Y90 (SIRveNIB)** (Yttrium-90 resin microspheres)	Radiation	1.12 (0.88–1.42)	SIRveNIB trial, locoregional selective internal radiation therapy (SIRT)	III (approved for colon cancer)	NCT01135056 [56]
**Y90 (SARAH)**	Radiation	1.15 (0.94–1.41)	Sorafenib vs. Radioembolization in Advanced Hepatocellular carcinoma (SARAH) trial, yttrium-90 (Y-90) resin microspheres	III	[57,58,59]
**2nd line treatment (FDA-approved and Phase III investigations in advanced HCC)**					
**Regorafenib**	VEGFR1-3, RET, KIT PDGFRa/b. FGFR1/2. Raf	Regorafenib 0.63 (0.50–0.79)		Approved	NCT01774344 [60]
**Ramucirumab**	VEGFR2	0.71 (0.53–0.95)	In advanced HCC with AFP > 400 ng/mL	Approved	NCT02435433
**Cabozantinib**	EGFR2, MET, RON, RET, TIE2, TAMkinases	0.76 (0.63–0.92)	CELESTIAL trial, HCC progression on prior sorafenib	Approved	NCT01908426 [61]
**Pembrolizumab**	PD-1	0.78 (0.61–0.99)	1 Q3W + BSC 8.3%	Approved	NCT02702401 [62]
**Brivanib**	PD-1	0.89 (0.69–1.15)	BRISK-FL study	III	NCT00858871 [55]; NCT00825955 [63]
**Tivantinib**	MET	0.97 (0.75–1.25)	METIV-HCC	III	NCT01755767 [64]
**Everolimus**	mTOR (mTORC1 complex)	1.95 (0.86–1.27)	EVOLVE-1	III	NCT01035229 [65]
**Sorafenib**	VEGFR1-3, Raf, PDGFRb, KIT, Fit-3	0.69 (0.55 to 0.87)	Sorafenib HCC Assessment Randomized Protocol (SHARP) trial	Approved	NCT00105443 [66,67]
**Nivolumab ±** **ipilimumab**	PD-1 and CTLA-4		Neoadjuvant	II	NCT03222076

* Current systemic therapies for HCC: approved in unselected populations [40].

**Table 2:** Therapeutic targets for iCCA [49,75].

Therapy	Target	OR (HR)	Prevalence in iCCA	Note	Clinical Phases [49]
Ramucirumab	VEGFR2	0.710 (0·531–0·949)		AFP > 400 ng/mL, 2nd line, REACH-2	NCT01140347 [76]
Pemigatinib	FGFR2 fusion (EGFR G179A. exon18)		11–15%	FIGHT-302, Comparator (Gemcitabine + cisplatin)	NCT03656536 [75]
Fisogatinib (BLU-554), Ivosidenib	IDH1 mutation		5–13%		NCT02989857, NCT02273739 [40,41]
	MET amplification		2–6%		
			2–3%	MSI high TMB high	
	FGFR2 V564F, N564				
Dabrafenib (BRAFi) + Trametinib (MEFi)	BRAF600E gain of function mutations		5% (BRAF)		
Larotrectinib	TRK Fusion			TRK fusion–positive cancer	ClinicalTrials.gov numbers, NCT02122913, NCT02637687, and NCT02576431 [74]
Futibatinib (TAS-120)	irreversible FGFR1–4 inhibitor			Comparator (Gemcitabine + cisplatin)	III, NCT04093362 [77]; I NCT02052778
Infigratinib (BGJ398)	FGFR1–3			Comparator (Gemcitabine + cisplatin)	III, NCT03773302
Derazantinib (ARQ-087)	pan-FGFR				III, NCT03230318 NCT04087876

## References

[1] Barbara L., Benzi G., Gaiani S., Fusconi F., Zironi G., Siringo S., Rigamonti A., Barbara C., Grigioni W., Mazziotti A. (1992). Natural history of small untreated hepatocellular carcinoma in cirrhosis: A multivariate analysis of prognostic factors of tumor growth rate and patient survival. Hepatology.

[2] Ebara M., Ohto M., Shinagawa T., Sugiura N., Kimura K., Matsutani S., Morita M., Saisho H., Tsuchiya Y., Okuda K. (1986). Natural history of minute hepatocellular carcinoma smaller than three centimeters complicating cirrhosis. A study in 22 patients. Gastroenterology.

[3] El-Serag H.B., Mason A.C. (1999). Rising incidence of hepatocellular carcinoma in the United States. N. Engl. J. Med..

[4] Liang T.J., Heller T. (2004). Pathogenesis of hepatitis C-associated hepatocellular carcinoma. Gastroenterology.

[5] Okuda K. (2000). Hepatocellular carcinoma. J. Hepatol..

[6] Kanda M., Tateishi R., Yoshida H., Sato T., Masuzaki R., Ohki T., Imamura J., Goto T., Yoshida H., Hamamura K. (2008). Extrahepatic metastasis of hepatocellular carcinoma: Incidence and risk factors. Liver Int..

[7] Louis P., Scott K.P., Duncan S.H., Flint H.J. (2007). Understanding the effects of diet on bacterial metabolism in the large intestine. J. Appl. Microbiol..

[8] Nakamura Y., Mizuguchi T., Tanimizu N., Ichinohe N., Ooe H., Kawamoto M., Meguro M., Hirata K., Mitaka T. (2014). Preoperative hepatocyte transplantation improves the survival of rats with nonalcoholic steatohepatitis-related cirrhosis after partial hepatectomy. Cell Transplant..

[9] Shah S.A., Smith J.K., Li Y., Ng S.C., Carroll J.E., Tseng J.F. (2011). Underutilization of therapy for hepatocellular carcinoma in the medicare population. Cancer.

[10] Sonnenday C.J., Dimick J.B., Schulick R.D., Choti M.A. (2007). Racial and geographic disparities in the utilization of surgical therapy for hepatocellular carcinoma. J. Gastrointest. Surg..

[11] McClain C.J., Barve S., Deaciuc I., Kugelmas M., Hill D. (1999). Cytokines in alcoholic liver disease. Semin. Liver Dis..

[12] Massague J., Obenauf A.C. (2016). Metastatic colonization by circulating tumour cells. Nature.

[13] Kim J., Woo A.J., Chu J., Snow J.W., Fujiwara Y., Kim C.G., Cantor A.B., Orkin S.H. (2010). A Myc network accounts for similarities between embryonic stem and cancer cell transcription programs. Cell.

[14] Ikushima H., Todo T., Ino Y., Takahashi M., Saito N., Miyazawa K., Miyazono K. (2011). Glioma-initiating cells retain their tumorigenicity through integration of the Sox axis and Oct4 protein. J. Biol. Chem..

[15] Alison M.R. (2005). Liver stem cells: Implications for hepatocarcinogenesis. Stem. Cell Rev..

[16] Roskams T. (2006). Liver stem cells and their implication in hepatocellular and cholangiocarcinoma. Oncogene.

[17] Zender L., Spector M.S., Xue W., Flemming P., Cordon-Cardo C., Silke J., Fan S.T., Luk J.M., Wigler M., Hannon G.J. (2006). Identification and validation of oncogenes in liver cancer using an integrative oncogenomic approach. Cell.

[18] Tang Y., Kitisin K., Jogunoori W., Li C., Deng C.X., Mueller S.C., Ressom H.W., Rashid A., He A.R., Mendelson J.S. (2008). Progenitor/stem cells give rise to liver cancer due to aberrant TGF-beta and IL-6 signaling. Proc. Natl. Acad. Sci. USA.

[19] Feldman D.E., Chen C., Punj V., Tsukamoto H., Machida K. (2012). Pluripotency factor-mediated expression of the leptin receptor (OB-R) links obesity to oncogenesis through tumor-initiating stem cells. Proc. Natl. Acad. Sci. USA.

[20] Dalile B., Van Oudenhove L., Vervliet B., Verbeke K. (2019). The role of short-chain fatty acids in microbiota-gut-brain communication. Nat. Rev. Gastroenterol. Hepatol..

[21] Valk-Lingbeek M.E., Bruggeman S.W., van Lohuizen M. (2004). Stem cells and cancer; the polycomb connection. Cell.

[22] Chambers I., Smith A. (2004). Self-renewal of teratocarcinoma and embryonic stem cells. Oncogene.

[23] Beachy P.A., Karhadkar S.S., Berman D.M. (2004). Tissue repair and stem cell renewal in carcinogenesis. Nature.

[24] Bajaj J.S., Hays R.A. (2019). Manipulation of the Gut-Liver Axis Using Microbiome Restoration Therapy in Primary Sclerosing Cholangitis. Am. J. Gastroenterol..

[25] Bajaj J.S., Kassam Z., Fagan A., Gavis E.A., Liu E., Cox I.J., Kheradman R., Heuman D., Wang J., Gurry T. (2017). Fecal microbiota transplant from a rational stool donor improves hepatic encephalopathy: A randomized clinical trial. Hepatology.

[26] Cai X., Chiu Y.H., Chen Z.J. (2014). The cGAS-cGAMP-STING pathway of cytosolic DNA sensing and signaling. Mol. Cell.

[27] Gibb E.A., Warren R.L., Wilson G.W., Brown S.D., Robertson G.A., Morin G.B., Holt R.A. (2015). Activation of an endogenous retrovirus-associated long non-coding RNA in human adenocarcinoma. Genome Med..

[28] Llorente C., Schnabl B. (2015). The gut microbiota and liver disease. Cell Mol. Gastroenterol. Hepatol..

[29] Henao-Mejia J., Elinav E., Jin C., Hao L., Mehal W.Z., Strowig T., Thaiss C.A., Kau A.L., Eisenbarth S.C., Jurczak M.J. (2012). Inflammasome-mediated dysbiosis regulates progression of NAFLD and obesity. Nature.

[30] Rao A., Kosters A., Mells J.E., Zhang W., Setchell K.D., Amanso A.M., Wynn G.M., Xu T., Keller B.T., Yin H. (2016). Inhibition of ileal bile acid uptake protects against nonalcoholic fatty liver disease in high-fat diet-fed mice. Sci. Transl. Med..

[31] Tan F.P.Y., Beltranena E., Zijlstra R.T. (2021). Resistant starch: Implications of dietary inclusion on gut health and growth in pigs: A review. J. Anim. Sci. Biotechnol..

[32] Rountree C.B., Senadheera S., Mato J.M., Crooks G.M., Lu S.C. (2008). Expansion of liver cancer stem cells during aging in methionine adenosyltransferase 1A-deficient mice. Hepatology.

[33] Cicalese A., Bonizzi G., Pasi C.E., Faretta M., Ronzoni S., Giulini B., Brisken C., Minucci S., Di Fiore P.P., Pelicci P.G. (2009). The tumor suppressor p53 regulates polarity of self-renewing divisions in mammary stem cells. Cell.

[34] Knoblich J.A. (2010). Asymmetric cell division: Recent developments and their implications for tumour biology. Nat. Rev. Mol. Cell Biol..

[35] Martin-Belmonte F., Perez-Moreno M. (2012). Epithelial cell polarity, stem cells and cancer. Nat. Rev. Cancer.

[36] Den Besten G., van Eunen K., Groen A.K., Venema K., Reijngoud D.J., Bakker B.M. (2013). The role of short-chain fatty acids in the interplay between diet, gut microbiota, and host energy metabolism. J. Lipid Res..

[37] Jiang L., Lang S., Duan Y., Zhang X., Gao B., Chopyk J., Schwanemann L.K., Ventura-Cots M., Bataller R., Bosques-Padilla F. (2020). Intestinal Virome in Patients With Alcoholic Hepatitis. Hepatology.

[38] Lang S., Demir M., Martin A., Jiang L., Zhang X., Duan Y., Gao B., Wisplinghoff H., Kasper P., Roderburg C. (2020). Intestinal Virome Signature Associated With Severity of Nonalcoholic Fatty Liver Disease. Gastroenterology.

[39] Feldman D.E., Chen C., Punj V., Machida K. (2013). The TBC1D15 oncoprotein controls stem cell self-renewal through destabilization of the Numb-p53 complex. PLoS ONE.

[40] Llovet J.M., Montal R., Sia D., Finn R.S. (2018). Molecular therapies and precision medicine for hepatocellular carcinoma. Nat. Rev. Clin. Oncol..

[41] Zehir A., Benayed R., Shah R.H., Syed A., Middha S., Kim H.R., Srinivasan P., Gao J., Chakravarty D., Devlin S.M. (2017). Mutational landscape of metastatic cancer revealed from prospective clinical sequencing of 10,000 patients. Nat. Med..

[42] Cope K., Risby T., Diehl A.M. (2000). Increased gastrointestinal ethanol production in obese mice: Implications for fatty liver disease pathogenesis. Gastroenterology.

[43] Kwong T.N.Y., Wang X., Nakatsu G., Chow T.C., Tipoe T., Dai R.Z.W., Tsoi K.K.K., Wong M.C.S., Tse G., Chan M.T.V. (2018). Association Between Bacteremia From Specific Microbes and Subsequent Diagnosis of Colorectal Cancer. Gastroenterology.

[44] Brown J.M., Hazen S.L. (2015). The gut microbial endocrine organ: Bacterially derived signals driving cardiometabolic diseases. Annu. Rev. Med..

[45] Tanoue T., Morita S., Plichta D.R., Skelly A.N., Suda W., Sugiura Y., Narushima S., Vlamakis H., Motoo I., Sugita K. (2019). A defined commensal consortium elicits CD8 T cells and anti-cancer immunity. Nature.

[46] Schulze K., Imbeaud S., Letouze E., Alexandrov L.B., Calderaro J., Rebouissou S., Couchy G., Meiller C., Shinde J., Soysouvanh F. (2015). Exome sequencing of hepatocellular carcinomas identifies new mutational signatures and potential therapeutic targets. Nat. Genet.

[47] Castaneda F.E., Walia B., Vijay-Kumar M., Patel N.R., Roser S., Kolachala V.L., Rojas M., Wang L., Oprea G., Garg P. (2005). Targeted deletion of metalloproteinase 9 attenuates experimental colitis in mice: Central role of epithelial-derived MMP. Gastroenterology.

[48] Reynolds J.M., Pappu B.P., Peng J., Martinez G.J., Zhang Y., Chung Y., Ma L., Yang X.O., Nurieva R.I., Tian Q. (2010). Toll-like receptor 2 signaling in CD4(+) T lymphocytes promotes T helper 17 responses and regulates the pathogenesis of autoimmune disease. Immunity.

[49] Banales J.M., Marin J.J.G., Lamarca A., Rodrigues P.M., Khan S.A., Roberts L.R., Cardinale V., Carpino G., Andersen J.B., Braconi C. (2020). Cholangiocarcinoma 2020: The next horizon in mechanisms and management. Nat. Rev. Gastroenterol. Hepatol..

[50] Finn R.S., Qin S., Ikeda M., Galle P.R., Ducreux M., Kim T.Y., Kudo M., Breder V., Merle P., Kaseb A.O. (2020). Atezolizumab plus Bevacizumab in Unresectable Hepatocellular Carcinoma. N. Engl. J. Med..

[51] El-Khoueiry A.B., Sangro B., Yau T., Crocenzi T.S., Kudo M., Hsu C., Kim T.Y., Choo S.P., Trojan J., Welling T.H.R. (2017). Nivolumab in patients with advanced hepatocellular carcinoma (CheckMate 040): An open-label, non-comparative, phase 1/2 dose escalation and expansion trial. Lancet.

[52] Kudo M., Finn R.S., Qin S., Han K.H., Ikeda K., Piscaglia F., Baron A., Park J.W., Han G., Jassem J. (2018). Lenvatinib versus sorafenib in first-line treatment of patients with unresectable hepatocellular carcinoma: A randomised phase 3 non-inferiority trial. Lancet.

[53] Zhu A.X., Rosmorduc O., Evans T.R., Ross P.J., Santoro A., Carrilho F.J., Bruix J., Qin S., Thuluvath P.J., Llovet J.M. (2015). SEARCH: A phase III, randomized, double-blind, placebo-controlled trial of sorafenib plus erlotinib in patients with advanced hepatocellular carcinoma. J. Clin. Oncol..

[54] Cainap C., Qin S., Huang W.T., Chung I.J., Pan H., Cheng Y., Kudo M., Kang Y.K., Chen P.J., Toh H.C. (2015). Linifanib versus Sorafenib in patients with advanced hepatocellular carcinoma: Results of a randomized phase III trial. J. Clin. Oncol..

[55] Johnson P.J., Qin S., Park J.W., Poon R.T., Raoul J.L., Philip P.A., Hsu C.H., Hu T.H., Heo J., Xu J. (2013). Brivanib versus sorafenib as first-line therapy in patients with unresectable, advanced hepatocellular carcinoma: Results from the randomized phase III BRISK-FL study. J. Clin. Oncol..

[56] Chow P.K.H., Gandhi M., Tan S.B., Khin M.W., Khasbazar A., Ong J., Choo S.P., Cheow P.C., Chotipanich C., Lim K. (2018). SIRveNIB: Selective Internal Radiation Therapy Versus Sorafenib in Asia-Pacific Patients with Hepatocellular Carcinoma. J. Clin. Oncol..

[57] Wang E.A., Stein J.P., Bellavia R.J., Broadwell S.R. (2017). Treatment options for unresectable HCC with a focus on SIRT with Yttrium-90 resin microspheres. Int. J. Clin. Pract..

[58] Golfieri R., Bilbao J.I., Carpanese L., Cianni R., Gasparini D., Ezziddin S., Paprottka P.M., Fiore F., Cappelli A., Rodriguez M. (2013). Comparison of the survival and tolerability of radioembolization in elderly vs. younger patients with unresectable hepatocellular carcinoma. J. Hepatol..

[59] Sangro B., Carpanese L., Cianni R., Golfieri R., Gasparini D., Ezziddin S., Paprottka P.M., Fiore F., Van Buskirk M., Bilbao J.I. (2011). Survival after yttrium-90 resin microsphere radioembolization of hepatocellular carcinoma across Barcelona clinic liver cancer stages: A European evaluation. Hepatology.

[60] Bruix J., Qin S., Merle P., Granito A., Huang Y.H., Bodoky G., Pracht M., Yokosuka O., Rosmorduc O., Breder V. (2017). Regorafenib for patients with hepatocellular carcinoma who progressed on sorafenib treatment (RESORCE): A randomised, double-blind, placebo-controlled, phase 3 trial. Lancet.

[61] Abou-Alfa G.K., Meyer T., Cheng A.L., El-Khoueiry A.B., Rimassa L., Ryoo B.Y., Cicin I., Merle P., Chen Y., Park J.W. (2018). Cabozantinib in Patients with Advanced and Progressing Hepatocellular Carcinoma. N. Engl. J. Med..

[62] Finn R.S., Ryoo B.Y., Merle P., Kudo M., Bouattour M., Lim H.Y., Breder V., Edeline J., Chao Y., Ogasawara S. (2020). Pembrolizumab As Second-Line Therapy in Patients with Advanced Hepatocellular Carcinoma in KEYNOTE-240: A Randomized, Double-Blind, Phase III Trial. J. Clin. Oncol..

[63] Llovet J.M., Decaens T., Raoul J.L., Boucher E., Kudo M., Chang C., Kang Y.K., Assenat E., Lim H.Y., Boige V. (2013). Brivanib in patients with advanced hepatocellular carcinoma who were intolerant to sorafenib or for whom sorafenib failed: Results from the randomized phase III BRISK-PS study. J. Clin. Oncol..

[64] Rimassa L., Porta C., Borbath I., Daniele B., Finn R.S., Raoul J.L., Schwartz L.H., He A.R., Trojan J., Peck-Radosavljevic M. (2014). Tivantinib in MET-high hepatocellular carcinoma patients and the ongoing Phase III clinical trial. Hepat. Oncol..

[65] Zhu A.X., Kudo M., Assenat E., Cattan S., Kang Y.K., Lim H.Y., Poon R.T., Blanc J.F., Vogel A., Chen C.L. (2014). Effect of everolimus on survival in advanced hepatocellular carcinoma after failure of sorafenib: The EVOLVE-1 randomized clinical trial. JAMA.

[66] Llovet J.M., Ricci S., Mazzaferro V., Hilgard P., Gane E., Blanc J.F., de Oliveira A.C., Santoro A., Raoul J.L., Forner A. (2008). Sorafenib in advanced hepatocellular carcinoma. N. Engl. J. Med..

[67] Llovet J.M., Pena C.E., Lathia C.D., Shan M., Meinhardt G., Bruix J., Group S.I.S. (2012). Plasma biomarkers as predictors of outcome in patients with advanced hepatocellular carcinoma. Clin. Cancer Res..

[68] Ribas A. (2012). Tumor immunotherapy directed at PD-1. N. Engl. J. Med..

[69] Wallin J.J., Bendell J.C., Funke R., Sznol M., Korski K., Jones S., Hernandez G., Mier J., He X., Hodi F.S. (2016). Atezolizumab in combination with bevacizumab enhances antigen-specific T-cell migration in metastatic renal cell carcinoma. Nat. Commun..

[70] Hegde P.S., Wallin J.J., Mancao C. (2018). Predictive markers of anti-VEGF and emerging role of angiogenesis inhibitors as immunotherapeutics. Semin. Cancer Biol..

[71] Finn R.S., Ikeda M., Zhu A.X., Sung M.W., Baron A.D., Kudo M., Okusaka T., Kobayashi M., Kumada H., Kaneko S. (2020). Phase Ib Study of Lenvatinib Plus Pembrolizumab in Patients With Unresectable Hepatocellular Carcinoma. J. Clin. Oncol..

[72] Mellman I., Coukos G., Dranoff G. (2011). Cancer immunotherapy comes of age. Nature.

[73] Sangro B., Gomez-Martin C., de la Mata M., Inarrairaegui M., Garralda E., Barrera P., Riezu-Boj J.I., Larrea E., Alfaro C., Sarobe P. (2013). A clinical trial of CTLA-4 blockade with tremelimumab in patients with hepatocellular carcinoma and chronic hepatitis C. J. Hepatol..

[74] Drilon A., Laetsch T.W., Kummar S., DuBois S.G., Lassen U.N., Demetri G.D., Nathenson M., Doebele R.C., Farago A.F., Pappo A.S. (2018). Efficacy of Larotrectinib in TRK Fusion-Positive Cancers in Adults and Children. N. Engl. J. Med..

[75] Bekaii-Saab T.S., Valle J.W., Cutsem E.V., Rimassa L., Furuse J., Ioka T., Melisi D., Macarulla T., Bridgewater J., Wasan H. (2020). FIGHT-302: First-line pemigatinib vs gemcitabine plus cisplatin for advanced cholangiocarcinoma with FGFR2 rearrangements. Future Oncol..

[76] Zhu A.X., Kang Y.K., Yen C.J., Finn R.S., Galle P.R., Llovet J.M., Assenat E., Brandi G., Pracht M., Lim H.Y. (2019). Ramucirumab after sorafenib in patients with advanced hepatocellular carcinoma and increased alpha-fetoprotein concentrations (REACH-2): A randomised, double-blind, placebo-controlled, phase 3 trial. Lancet Oncol..

[77] Goyal L., Shi L., Liu L.Y., Fece de la Cruz F., Lennerz J.K., Raghavan S., Leschiner I., Elagina L., Siravegna G., Ng R.W.S. (2019). TAS-120 Overcomes Resistance to ATP-Competitive FGFR Inhibitors in Patients with FGFR2 Fusion-Positive Intrahepatic Cholangiocarcinoma. Cancer Discov..

[78] Sivan A., Corrales L., Hubert N., Williams J.B., Aquino-Michaels K., Earley Z.M., Benyamin F.W., Lei Y.M., Jabri B., Alegre M.L. (2015). Commensal Bifidobacterium promotes antitumor immunity and facilitates anti-PD-L1 efficacy. Science.

[79] Atarashi K., Tanoue T., Shima T., Imaoka A., Kuwahara T., Momose Y., Cheng G., Yamasaki S., Saito T., Ohba Y. (2011). Induction of colonic regulatory T cells by indigenous Clostridium species. Science.

[80] Routy B., Le Chatelier E., Derosa L., Duong C.P.M., Alou M.T., Daillere R., Fluckiger A., Messaoudene M., Rauber C., Roberti M.P. (2018). Gut microbiome influences efficacy of PD-1-based immunotherapy against epithelial tumors. Science.

[81] Frankel A.E., Coughlin L.A., Kim J., Froehlich T.W., Xie Y., Frenkel E.P., Koh A.Y. (2017). Metagenomic Shotgun Sequencing and Unbiased Metabolomic Profiling Identify Specific Human Gut Microbiota and Metabolites Associated with Immune Checkpoint Therapy Efficacy in Melanoma Patients. Neoplasia.

[82] Gopalakrishnan V., Spencer C.N., Nezi L., Reuben A., Andrews M.C., Karpinets T.V., Prieto P.A., Vicente D., Hoffman K., Wei S.C. (2018). Gut microbiome modulates response to anti-PD-1 immunotherapy in melanoma patients. Science.

[83] Matson V., Fessler J., Bao R., Chongsuwat T., Zha Y., Alegre M.L., Luke J.J., Gajewski T.F. (2018). The commensal microbiome is associated with anti-PD-1 efficacy in metastatic melanoma patients. Science.

[84] Chung W.S., Walker A.W., Louis P., Parkhill J., Vermeiren J., Bosscher D., Duncan S.H., Flint H.J. (2016). Modulation of the human gut microbiota by dietary fibres occurs at the species level. BMC Biol..

[85] Vetizou M., Pitt J.M., Daillere R., Lepage P., Waldschmitt N., Flament C., Rusakiewicz S., Routy B., Roberti M.P., Duong C.P. (2015). Anticancer immunotherapy by CTLA-4 blockade relies on the gut microbiota. Science.

[86] Nigar S., Shimosato T. (2019). Cooperation of Oligodeoxynucleotides and Synthetic Molecules as Enhanced Immune Modulators. Front. Nutr..

[87] Pascual-Itoiz M.A., Pena-Cearra A., Martin-Ruiz I., Lavin J.L., Simo C., Rodriguez H., Atondo E., Flores J.M., Carreras-Gonzalez A., Tomas-Cortazar J. (2020). The mitochondrial negative regulator MCJ modulates the interplay between microbiota and the host during ulcerative colitis. Sci. Rep..

[88] Rowan-Nash A.D., Korry B.J., Mylonakis E., Belenky P. (2019). Cross-Domain and Viral Interactions in the Microbiome. Microbiol. Mol. Biol. Rev..

[89] Lang S., Schnabl B. (2020). Microbiota and Fatty Liver Disease-the Known, the Unknown, and the Future. Cell Host Microbe.

[90] Lourenco M., Chaffringeon L., Lamy-Besnier Q., Pedron T., Campagne P., Eberl C., Berard M., Stecher B., Debarbieux L., De Sordi L. (2020). The Spatial Heterogeneity of the Gut Limits Predation and Fosters Coexistence of Bacteria and Bacteriophages. Cell Host Microbe.

[91] Laohaviroj M., Potriquet J., Jia X., Suttiprapa S., Chamgramol Y., Pairojkul C., Sithithaworn P., Mulvenna J., Sripa B. (2017). A comparative proteomic analysis of bile for biomarkers of cholangiocarcinoma. Tumour Biol..

[92] Ramirez-Farias C., Slezak K., Fuller Z., Duncan A., Holtrop G., Louis P. (2009). Effect of inulin on the human gut microbiota: Stimulation of Bifidobacterium adolescentis and Faecalibacterium prausnitzii. Br. J. Nutr..

[93] Lopez-Siles M., Martinez-Medina M., Suris-Valls R., Aldeguer X., Sabat-Mir M., Duncan S.H., Flint H.J., Garcia-Gil L.J. (2016). Changes in the Abundance of Faecalibacterium prausnitzii Phylogroups I and II in the Intestinal Mucosa of Inflammatory Bowel Disease and Patients with Colorectal Cancer. Inflamm. Bowel Dis..

[94] Benus R.F., van der Werf T.S., Welling G.W., Judd P.A., Taylor M.A., Harmsen H.J., Whelan K. (2010). Association between Faecalibacterium prausnitzii and dietary fibre in colonic fermentation in healthy human subjects. Br. J. Nutr..

[95] Hooda S., Boler B.M., Serao M.C., Brulc J.M., Staeger M.A., Boileau T.W., Dowd S.E., Fahey G.C., Swanson K.S. (2012). 454 pyrosequencing reveals a shift in fecal microbiota of healthy adult men consuming polydextrose or soluble corn fiber. J. Nutr..

[96] Licht T.R., Hansen M., Bergstrom A., Poulsen M., Krath B.N., Markowski J., Dragsted L.O., Wilcks A. (2010). Effects of apples and specific apple components on the cecal environment of conventional rats: Role of apple pectin. BMC Microbiol..

[97] Lopez-Siles M., Khan T.M., Duncan S.H., Harmsen H.J., Garcia-Gil L.J., Flint H.J. (2012). Cultured representatives of two major phylogroups of human colonic Faecalibacterium prausnitzii can utilize pectin, uronic acids, and host-derived substrates for growth. Appl. Environ. Microbiol..

[98] Salvatore S., Heuschkel R., Tomlin S., Davies S.E., Edwards S., Walker-Smith J.A., French I., Murch S.H. (2000). A pilot study of N-acetyl glucosamine, a nutritional substrate for glycosaminoglycan synthesis, in paediatric chronic inflammatory bowel disease. Aliment. Pharmacol. Ther..

[99] Sadaghian Sadabad M., von Martels J.Z., Khan M.T., Blokzijl T., Paglia G., Dijkstra G., Harmsen H.J., Faber K.N. (2015). A simple coculture system shows mutualism between anaerobic faecalibacteria and epithelial Caco-2 cells. Sci. Rep..

[100] Rafter J., Bennett M., Caderni G., Clune Y., Hughes R., Karlsson P.C., Klinder A., O’Riordan M., O’Sullivan G.C., Pool-Zobel B. (2007). Dietary synbiotics reduce cancer risk factors in polypectomized and colon cancer patients. Am. J. Clin. Nutr..

[101] Ernst C.M., Staubitz P., Mishra N.N., Yang S.J., Hornig G., Kalbacher H., Bayer A.S., Kraus D., Peschel A. (2009). The bacterial defensin resistance protein MprF consists of separable domains for lipid lysinylation and antimicrobial peptide repulsion. PLoS Pathog..

[102] Ma H.Y., Xu J., Liu X., Zhu Y., Gao B., Karin M., Tsukamoto H., Jeste D.V., Grant I., Roberts A.J. (2016). The role of IL-17 signaling in regulation of the liver-brain axis and intestinal permeability in Alcoholic Liver Disease. Curr. Pathobiol. Rep..

[103] Van den Broek L.A., Lloyd R.M., Beldman G., Verdoes J.C., McCleary B.V., Voragen A.G. (2005). Cloning and characterization of arabinoxylan arabinofuranohydrolase-D3 (AXHd3) from Bifidobacterium adolescentis DSM20083. Appl. Microbiol. Biotechnol..

[104] Rios-Covian D., Arboleya S., Hernandez-Barranco A.M., Alvarez-Buylla J.R., Ruas-Madiedo P., Gueimonde M., de los Reyes-Gavilan C.G. (2013). Interactions between Bifidobacterium and Bacteroides species in cofermentations are affected by carbon sources, including exopolysaccharides produced by bifidobacteria. Appl. Environ. Microbiol..

[105] Koszewicz M., Jaroch J., Brzecka A., Ejma M., Budrewicz S., Mikhaleva L.M., Muresanu C., Schield P., Somasundaram S.G., Kirkland C.E. (2021). Dysbiosis is one of the risk factor for stroke and cognitive impairment and potential target for treatment. Pharmacol. Res..

[106] Pitt J.M., Vetizou M., Gomperts Boneca I., Lepage P., Chamaillard M., Zitvogel L. (2017). Enhancing the clinical coverage and anticancer efficacy of immune checkpoint blockade through manipulation of the gut microbiota. Oncoimmunology.

[107] Heinken A., Khan M.T., Paglia G., Rodionov D.A., Harmsen H.J., Thiele I. (2014). Functional metabolic map of Faecalibacterium prausnitzii, a beneficial human gut microbe. J. Bacteriol..

[108] Kamath P.S., Wiesner R.H., Malinchoc M., Kremers W., Therneau T.M., Kosberg C.L., D’Amico G., Dickson E.R., Kim W.R. (2001). A model to predict survival in patients with end-stage liver disease. Hepatology.

[109] Frosali S., Pagliari D., Gambassi G., Landolfi R., Pandolfi F., Cianci R. (2015). How the Intricate Interaction among Toll-Like Receptors, Microbiota, and Intestinal Immunity Can Influence Gastrointestinal Pathology. J. Immunol. Res..

[110] Spiljar M., Merkler D., Trajkovski M. (2017). The Immune System Bridges the Gut Microbiota with Systemic Energy Homeostasis: Focus on TLRs, Mucosal Barrier, and SCFAs. Front. Immunol..

[111] Malik S., Sadhu S., Elesela S., Pandey R.P., Chawla A.S., Sharma D., Panda L., Rathore D., Ghosh B., Ahuja V. (2017). Transcription factor Foxo1 is essential for IL-9 induction in T helper cells. Nat. Commun..

[112] Negi V., Paul D., Das S., Bajpai P., Singh S., Mukhopadhyay A., Agrawal A., Ghosh B. (2015). Altered expression and editing of miRNA-100 regulates iTreg differentiation. Nucleic Acids Res..

[113] Atarashi K., Tanoue T., Ando M., Kamada N., Nagano Y., Narushima S., Suda W., Imaoka A., Setoyama H., Nagamori T. (2015). Th17 Cell Induction by Adhesion of Microbes to Intestinal Epithelial Cells. Cell.

[114] Goto Y., Panea C., Nakato G., Cebula A., Lee C., Diez M.G., Laufer T.M., Ignatowicz L., Ivanov I.I. (2014). Segmented filamentous bacteria antigens presented by intestinal dendritic cells drive mucosal Th17 cell differentiation. Immunity.

[115] Wu W., Liu H.P., Chen F., Liu H., Cao A.T., Yao S., Sun M., Evans-Marin H.L., Zhao Y., Zhao Q. (2016). Commensal A4 bacteria inhibit intestinal Th2-cell responses through induction of dendritic cell TGF-beta production. Eur. J. Immunol..

[116] Atarashi K., Tanoue T., Oshima K., Suda W., Nagano Y., Nishikawa H., Fukuda S., Saito T., Narushima S., Hase K. (2013). Treg induction by a rationally selected mixture of Clostridia strains from the human microbiota. Nature.

[117] Hrncir T., Stepankova R., Kozakova H., Hudcovic T., Tlaskalova-Hogenova H. (2008). Gut microbiota and lipopolysaccharide content of the diet influence development of regulatory T cells: Studies in germ-free mice. BMC Immunol..

[118] Telesford K.M., Yan W., Ochoa-Reparaz J., Pant A., Kircher C., Christy M.A., Begum-Haque S., Kasper D.L., Kasper L.H. (2015). A commensal symbiotic factor derived from Bacteroides fragilis promotes human CD39(+)Foxp3(+) T cells and Treg function. Gut Microbes.

[119] Neff C.P., Rhodes M.E., Arnolds K.L., Collins C.B., Donnelly J., Nusbacher N., Jedlicka P., Schneider J.M., McCarter M.D., Shaffer M. (2016). Diverse Intestinal Bacteria Contain Putative Zwitterionic Capsular Polysaccharides with Anti-inflammatory Properties. Cell Host Microbe.

[120] Stingele F., Corthesy B., Kusy N., Porcelli S.A., Kasper D.L., Tzianabos A.O. (2004). Zwitterionic polysaccharides stimulate T cells with no preferential V beta usage and promote anergy, resulting in protection against experimental abscess formation. J. Immunol..

[121] Dasgupta S., Erturk-Hasdemir D., Ochoa-Reparaz J., Reinecker H.C., Kasper D.L. (2014). Plasmacytoid dendritic cells mediate anti-inflammatory responses to a gut commensal molecule via both innate and adaptive mechanisms. Cell Host Microbe.

[122] Mazmanian S.K., Liu C.H., Tzianabos A.O., Kasper D.L. (2005). An immunomodulatory molecule of symbiotic bacteria directs maturation of the host immune system. Cell.

[123] Arumugam M., Raes J., Pelletier E., Le Paslier D., Yamada T., Mende D.R., Fernandes G.R., Tap J., Bruls T., Batto J.M. (2011). Enterotypes of the human gut microbiome. Nature.

[124] Qin J., Li R., Raes J., Arumugam M., Burgdorf K.S., Manichanh C., Nielsen T., Pons N., Levenez F., Yamada T. (2010). A human gut microbial gene catalogue established by metagenomic sequencing. Nature.

[125] Tzianabos A.O., Pantosti A., Baumann H., Brisson J.R., Jennings H.J., Kasper D.L. (1992). The capsular polysaccharide of Bacteroides fragilis comprises two ionically linked polysaccharides. J. Biol. Chem..

[126] Cebula A., Seweryn M., Rempala G.A., Pabla S.S., McIndoe R.A., Denning T.L., Bry L., Kraj P., Kisielow P., Ignatowicz L. (2013). Thymus-derived regulatory T cells contribute to tolerance to commensal microbiota. Nature.

[127] Sonnenburg J.L., Chen C.T., Gordon J.I. (2006). Genomic and metabolic studies of the impact of probiotics on a model gut symbiont and host. PLoS Biol..

[128] Yuan J., Chen C., Cui J., Lu J., Yan C., Wei X., Zhao X., Li N., Li S., Xue G. (2019). Fatty Liver Disease Caused by High-Alcohol-Producing Klebsiella pneumoniae. Cell Metab..

[129] Belkaid Y., Hand T.W. (2014). Role of the microbiota in immunity and inflammation. Cell.

[130] Llopis M., Cassard A.M., Wrzosek L., Boschat L., Bruneau A., Ferrere G., Puchois V., Martin J.C., Lepage P., Le Roy T. (2016). Intestinal microbiota contributes to individual susceptibility to alcoholic liver disease. Gut.

[131] Chatterjee A., Johnson C.N., Luong P., Hullahalli K., McBride S.W., Schubert A.M., Palmer K.L., Carlson P.E., Duerkop B.A. (2019). Bacteriophage Resistance Alters Antibiotic-Mediated Intestinal Expansion of Enterococci. Infect. Immun..

[132] Duan Y., Llorente C., Lang S., Brandl K., Chu H., Jiang L., White R.C., Clarke T.H., Nguyen K., Torralba M. (2019). Bacteriophage targeting of gut bacterium attenuates alcoholic liver disease. Nature.

[133] Woodhouse C.A., Patel V.C., Singanayagam A., Shawcross D.L. (2018). Review article: The gut microbiome as a therapeutic target in the pathogenesis and treatment of chronic liver disease. Aliment Pharmacol. Ther..

[134] Qin N., Yang F., Li A., Prifti E., Chen Y., Shao L., Guo J., Le Chatelier E., Yao J., Wu L. (2014). Alterations of the human gut microbiome in liver cirrhosis. Nature.

[135] Kim H.J., Lee J., Choi J.H., Bahinski A., Ingber D.E. (2016). Co-culture of Living Microbiome with Microengineered Human Intestinal Villi in a Gut-on-a-Chip Microfluidic Device. J. Vis. Exp..

[136] De Gregorio V., Telesco M., Corrado B., Rosiello V., Urciuolo F., Netti P.A., Imparato G. (2020). Intestine-Liver Axis On-Chip Reveals the Intestinal Protective Role on Hepatic Damage by Emulating Ethanol First-Pass Metabolism. Front. Bioeng. Biotechnol..

[137] Kasendra M., Luc R., Yin J., Manatakis D.V., Kulkarni G., Lucchesi C., Sliz J., Apostolou A., Sunuwar L., Obrigewitch J. (2020). Duodenum Intestine-Chip for preclinical drug assessment in a human relevant model. eLife.

[138] Grassart A., Malarde V., Gobaa S., Sartori-Rupp A., Kerns J., Karalis K., Marteyn B., Sansonetti P., Sauvonnet N. (2019). Bioengineered Human Organ-on-Chip Reveals Intestinal Microenvironment and Mechanical Forces Impacting Shigella Infection. Cell Host Microbe.

[139] Jang K.J., Otieno M.A., Ronxhi J., Lim H.K., Ewart L., Kodella K.R., Petropolis D.B., Kulkarni G., Rubins J.E., Conegliano D. (2019). Reproducing human and cross-species drug toxicities using a Liver-Chip. Sci. Transl. Med..

[140] Miller T.L., Wolin M.J. (1996). Pathways of acetate, propionate, and butyrate formation by the human fecal microbial flora. Appl. Environ. Microbiol..

[141] Bergman E.N. (1990). Energy contributions of volatile fatty acids from the gastrointestinal tract in various species. Physiol. Rev..

[142] Macfarlane S., Macfarlane G.T. (2003). Regulation of short-chain fatty acid production. Proc. Nutr. Soc..

[143] Cummings J.H., Pomare E.W., Branch W.J., Naylor C.P., Macfarlane G.T. (1987). Short chain fatty acids in human large intestine, portal, hepatic and venous blood. Gut.

[144] Stumpff F. (2018). A look at the smelly side of physiology: Transport of short chain fatty acids. Pflug. Arch..

[145] Bajaj J.S., Khoruts A. (2020). Microbiota changes and intestinal microbiota transplantation in liver diseases and cirrhosis. J. Hepatol..

[146] Vrieze A., Van Nood E., Holleman F., Salojarvi J., Kootte R.S., Bartelsman J.F., Dallinga-Thie G.M., Ackermans M.T., Serlie M.J., Oozeer R. (2012). Transfer of intestinal microbiota from lean donors increases insulin sensitivity in individuals with metabolic syndrome. Gastroenterology.

[147] Craven L., Rahman A., Nair Parvathy S., Beaton M., Silverman J., Qumosani K., Hramiak I., Hegele R., Joy T., Meddings J. (2020). Allogenic Fecal Microbiota Transplantation in Patients With Nonalcoholic Fatty Liver Disease Improves Abnormal Small Intestinal Permeability: A Randomized Control Trial. Am. J. Gastroenterol..

[148] Liu R., Kang J.D., Sartor R.B., Sikaroodi M., Fagan A., Gavis E.A., Zhou H., Hylemon P.B., Herzog J.W., Li X. (2020). Neuroinflammation in Murine Cirrhosis Is Dependent on the Gut Microbiome and Is Attenuated by Fecal Transplant. Hepatology.

[149] Bajaj J.S., Salzman N., Acharya C., Takei H., Kakiyama G., Fagan A., White M.B., Gavis E.A., Holtz M.L., Hayward M. (2019). Microbial functional change is linked with clinical outcomes after capsular fecal transplant in cirrhosis. JCI Insight.

[150] Bajaj J.S., Gavis E.A., Fagan A., Wade J.B., Thacker L.R., Fuchs M., Patel S., Davis B., Meador J., Puri P. (2020). A Randomized Clinical Trial of Fecal Microbiota Transplant for Alcohol Use Disorder. Hepatology.

[151] Arfianti A., Pok S., Barn V., Haigh W.G., Yeh M.M., Ioannou G.N., Teoh N.C., Farrell G.C. (2020). Exercise retards hepatocarcinogenesis in obese mice independently of weight control. J. Hepatol..

